# Effects of Craniotomy and Endoscopic Endonasal Transsphenoidal Surgery on Bodyweight in Adult-Onset Craniopharyngioma: A Single-Center Retrospective Study

**DOI:** 10.3390/jcm12041578

**Published:** 2023-02-16

**Authors:** Yanbin Li, Youchao Xiao, Wentao Wu, Lu Jin, Yanfei Jia, Kefan Cai, Ning Qiao, Lei Cao, Songbai Gui

**Affiliations:** Department of Neurosurgery, Beijing Tiantan Hospital, Capital Medical University, Beijing 100070, China

**Keywords:** adult-onset craniopharyngioma, endoscopic endonasal transsphenoidal surgery, hypothalamic involvement, weight control, endocrine function

## Abstract

Craniopharyngioma (CP) is a histologically benign tumor with high mortality and morbidity. Although surgical treatment is essential in managing CP, the best surgical approach is debated. A retrospective cohort of 117 patients with adult-onset CP (AOCP) treated between 2018 and 2020 in Beijing Tiantan Hospital was identified and examined. The effects of traditional craniotomy (TC) and endoscopic endonasal transsphenoidal surgery (EETS) on the extent of surgical resection, hypothalamic involvement (HI), postoperative endocrine function, and postoperative weight were compared in the cohort. The cohort comprised 43 males and 74 females, divided into the TC (*n* = 59) and EETS (*n* = 58) groups. The EETS group possessed a higher rate of gross total resection (GTR) (adjusted odds ratio (aOR) = 4.08, *p* = 0.029) and improved HI (aOR = 2.58, *p* = 0.041) than the TC group. Worse postoperative HI was only observed in the TC group (5 patients). The EETS was associated with fewer adverse hormonal outcomes, including posterior pituitary dysfunction (aOR = 0.386, *p* = 0.040) and hypopituitarism (aOR = 0.384, *p* = 0.031). Additionally, multivariate logistic regression analysis confirmed that EETS was related to fewer cases of weight gain >5% (aOR = 0.376, *p* = 0.034), significant weight change (aOR = 0.379, *p* = 0.022), and postoperative obesity (aOR = 0.259, *p* = 0.032). Compared to TC, EETS shows advantages in accomplishing GTR, hypothalamus protection, postoperative endocrine function reservation, and postoperative weight control. These data suggest that the EETS deserves more application in managing patients with AOCP.

## 1. Introduction

Craniopharyngioma (CP), a histologically benign tumor originating from embryonic remnants of Rathke’s pouch, can occur in the intrasellar or suprasellar regions with a low incidence rate estimated at 0.5 to 2.5 new cases per million people annually [1,2]. Despite the significant advances in surgery, radiotherapy, and intracystic treatment, CP presents a unique challenge due to frequent recurrences and significant treatment-related morbidity [1,3]. Currently, the management of CP mainly involves surgery alone, irradiation alone, or a combination of both, although the optimal approach is still a subject of controversy [1]. Nonetheless, surgery plays a crucial role in treating patients with CP.

In managing CP, a surgical procedure allowing gross total resection (GTR) with less hypothalamic damage might be the most effective method for two reasons. First, the 2- and 5-year progression-free survival (PFS) rate of patients with CP is higher after GTR than after subgross total resection (STR) [4,5]. Second, hypothalamus-sparing is crucial to prevent damage-related sequelae, such as endocrine dysfunction, cognitive ability decline, and hypothalamic obesity.

Historically, traditional craniotomy (TC), including pterional, orbitozygomatic, sub-frontal, frontobasal interhemispheric, transpetrosal, and transcallosal-transventricular approaches, has been employed to resect CP [6,7,8]. In recent years, an increasing number of surgeons have been using endoscopic endonasal transsphenoidal surgery (EETS) in resecting CP due to improvements in equipment and refinements in EETS [9,10]. In patients with CP, the effects of EETS and TC have been compared in various items sufficiently, such as visual outcomes, endocrine function, cerebrospinal fluid leakage, recurrence rates, and postoperative seizures, but not postoperative weight [11,12,13,14]. Hypothalamic damage leads to endocrine dysfunction, temperature dysregulation, and obesity; obesity, in turn, reflects the severity of hypothalamic injury to a certain extent. Therefore, it is reasonable and critical to evaluate the effects of different operations on hypothalamus sparing by evaluating new-onset obesity following surgery.

This study focused on comparing hypothalamic obesity and endocrine dysfunction after EETS and TC surgeries to investigate the hypothalamic injury induced by both surgical approaches.

## 2. Patients and Methods

### 2.1. Patient Selection

**Figure 1:**
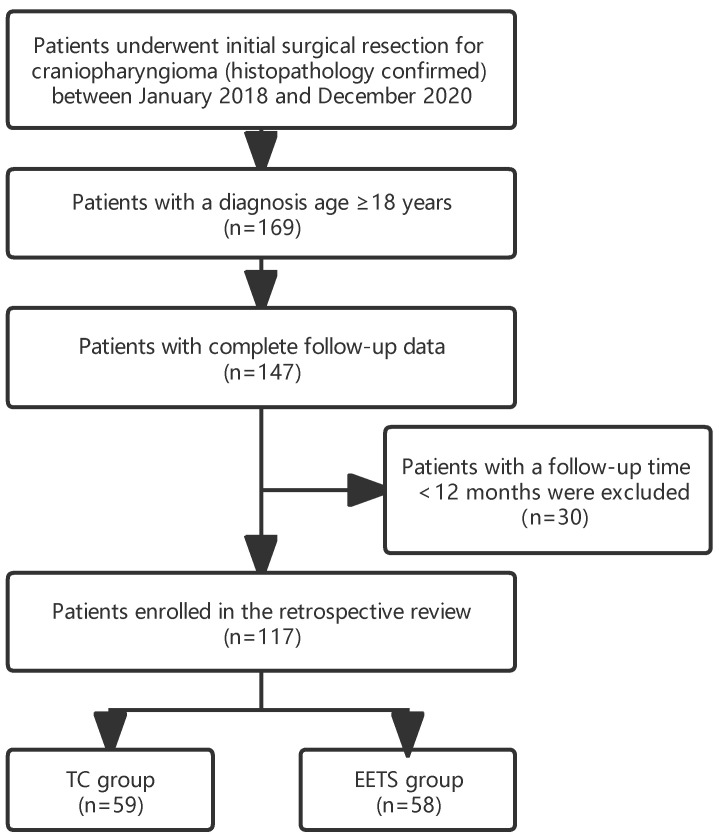
The flowchart of patient selection.

The Review Board of Beijing Tiantan Hospital approved the current study’s design (KY-2021-041-02), and written consent was obtained from all study subjects. We conducted a single-center, retrospective cohort study consisting of patients with adult-onset CP (AOCP) who underwent EETS or TC at Beijing Tiantan Hospital between January 2018 and December 2020, and the choice of surgical approach was determined based on the principles provided in the article [8] we published and the opinions of patients and their families. Four senior surgeons on the skull base surgery team performed all surgeries, and each senior surgeon performs more than 50 pituitary operations per year. Patients were included in the study if they met all of the following criteria: (I) Patients had not received radiotherapy or cyst aspiration before receiving initial surgery for CP in our hospital; (II) Histopathological tumor subtype (adamantinomatous versus papillary) was available and recorded; (III) the diagnosis age was ≥18 years; (IV) The length of follow-up ≥12 months. Finally, 117 patients were enrolled in the retrospective study, divided into the EETS group (*n* = 58) and the TC group (*n* = 59). The flowchart summarizing the enrollment strategy is shown in Figure 1.

### 2.2. Data Collection and Definition

The baseline characteristics and demographics were obtained from the hospital medical records. The data on age, sex, height, tumor size, tumor volume, tumor consistency (cystic or not), the extent of surgical resection, histopathology subtype, follow-up length, preoperative and postoperative weight, preoperative and postoperative body mass index (BMI), preoperative and postoperative hypothalamic involvement (HI), and preoperative and postoperative endocrine function were collected. And patients were followed from primary surgery until the date of secondary surgery, receiving radiotherapy, death, or 31 December 2021, whichever came first.

**Figure 2:**
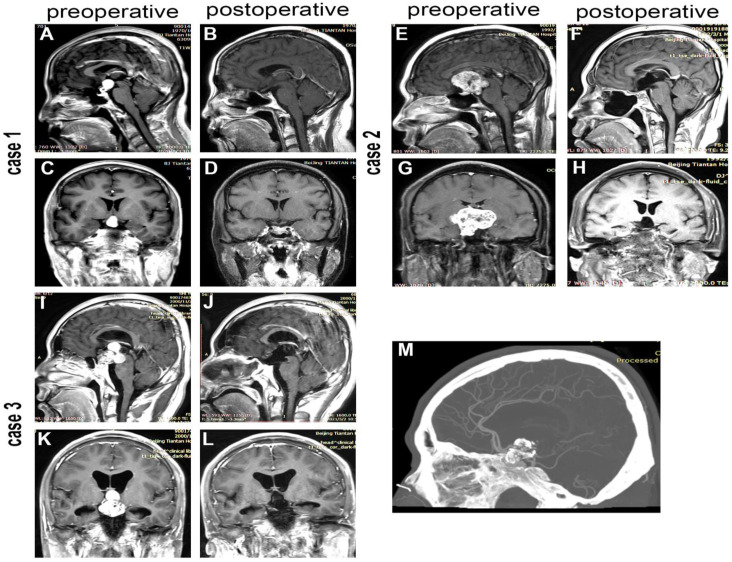
The application of EETS in resecting craniopharyngioma. Case 1 patient with preoperative hypothalamic involvement had improved hypothalamic involvement after EETS. Preoperative sagittal (**A**) and coronal (**C**) MR T1-weighted images showed tumor invasion of the hypothalamus; postoperative sagittal (**B**) and coronal (**D**) MR T1-weighted images showed the integrated hypothalamus. Case 2 patients with giant craniopharyngioma underwent gross total resection (GTR) using EETS. Preoperative sagittal (**E**) and coronal (**G**) MR T1-weighted images showed craniopharyngioma of giant size; postoperative sagittal (**F**) and coronal (**H**) MR T1-weighted images showed the GTR. Case 3 patients with calcified craniopharyngiomas underwent GTR using EETS. Preoperative sagittal (**I**), coronal (**K**) MR T1-weighted images, and preoperative sagittal (**M**) computed tomography images showed the craniopharyngioma possessed calcification; postoperative sagittal (**J**) and coronal (**L**) MR T1-weighted images showed the GTR.

The initial tumor size was characterized by the maximum diameter in three dimensions based on preoperative magnetic resonance imaging (MRI). Tumor volume was roughly calculated using the following formula: volume = 4/3 × π × (a/2 × b/2 × c/2), where a, b, and c are the maximum diameters of the tumor in each of the three dimensions [15]. Tumors were classified as cystic if more than 50% of the tumor volume was cystic [16]. The HI grade was identified using Puget’s grading system, which grades from 0 to 2 [17]. An improved HI converts from a higher grade preoperative HI to a lower grade postoperative HI (as shown in Figure 2, case 1), and a worse HI converts from a lower grade preoperative HI to a higher grade postoperative HI [18]. A postoperative brain MRI within three months after surgery was used to define postoperative HI. The extent of tumor resection was assessed by intraoperative observation and postoperative MRI, and GTR was defined as resection without visible remnant tumor on intraoperative assessment and postoperative MRI.

The criteria for endocrinological evaluation were consistent with those of a previous study [19]. Postoperative anterior pituitary function worsening was defined as (I) at least two new-onset anterior pituitary dysfunctions in patients with fewer than three pre-existing anterior pituitary dysfunctions or (II) one new-onset anterior pituitary dysfunction in patients with three pre-existing anterior pituitary dysfunctions. Postoperative posterior pituitary function worsening was defined as the occurrence of new-onset diabetes insipidus (DI). Postoperative hypopituitarism was regarded as posterior pituitary dysfunction combined with at least one anterior pituitary dysfunction simultaneously.

A BMI ≥ 30 kg/m^2^ indicates obesity, a BMI between 25 and 29.9 kg/m^2^ indicates overweight, and a BMI < 25 kg/m^2^ indicates a normal weight. The weight changes were computed using the following equation: weight change = (postoperative weight—preoperative weight)/preoperative weight. Consistent with previous studies [18,20], postoperative weight changes ≥ 5% were considered meaningful weight changes. Therefore, three clinical categories of weight change status were considered: weight loss > 5%, stable weight, and weight gain > 5%.

### 2.3. Statistical Analyses

We compared proportions between two and more groups using the Chi-square and Fisher exact tests. The paired t-test was used to analyze repeated measures within the same group, such as preoperative and postoperative BMI. Student’s *t*-test and Mann-Whitney U-test were used to analyze the parametric and nonparametric independent variables between groups, respectively. Univariate logistic regression analysis and multivariate logistic regression analysis were used to calculate the unadjusted odds ratio (uOR) and the adjusted odds ratio (aOR). Multivariate logistic regression analysis adjusted for confounding factors and identified the association between surgical modality (TC and EETS) and clinical outcomes. All statistical analyses were calculated using SSPS 17.0 (SSPS, Inc., Chicago, IL, USA) and GraphPad Prism 9.0 (GraphPad Software, Inc., La Jolla, CA, USA). In all analyses, a *p*-value < 0.05 was considered statistically significant.

## 3. Results

### 3.1. Patient Characteristics

As shown in Table 1, the EETS group included 21 males and 37 females, and the TC group included 22 males and 37 females. There were no significant differences between the EETS and TC groups concerning several characteristics, including mean age at diagnosis (42.84 years vs. 41.02 years), mean initial tumor size (3.10 cm vs. 2.89 cm), mean tumor volume (9.89 cm^3^ vs. 9.22 cm^3^), mean preoperative BMI (25.54 kg/m^2^ vs. 25.46 kg/m^2^), and median follow-up length (25.50 months vs. 26 months). In the EETS group, 65.5% of tumors were calcified compared to 66.1% in the TC group (*p* = 0.947). Moreover, the groups showed no significant difference in the proportion of cystic tumors (*p* = 0.450), the proportion of calcification (*p* = 0.947), the distributions of preoperative BMI (*p* = 0.569), and preoperative HI (*p* = 0.629).

### 3.2. Extent of Surgical Resection and Hypothalamus Involvement

As shown in Table 1, 99 patients underwent GTR and 18 patients underwent STR, respectively, and the EEA group possessed a higher rate of GTR than the TC group (91.4% vs. 78.0%, *p* = 0.027). Most patients in the entire cohort showed improved HI after surgery, while the TC group showed five patients with worse postoperative HI. The logistical analysis (Table 2) also confirmed that the rates of GTR (uOR = 3.82, *p* = 0.027; aOR = 4.08, *p* = 0.029) and improved HI (uOR = 1.93, *p* = 0.096; aOR = 2.58, *p* = 0.041) were higher in the EETS group. The patients with preoperative obesity acquired a lower rate of GTR than those with normal preoperative weight (aOR = 0.12, *p* = 0.03).

### 3.3. Hormonal Outcomes

**Figure 3:**
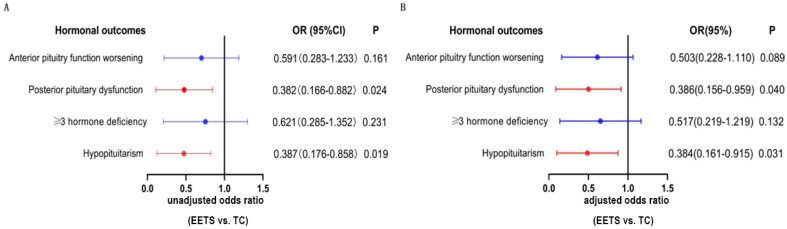
The association between the surgical method and hormonal outcomes. (**A**) The univariate logistic regression analysis; (**B**) The multivariate logistic regression analysis. The result showed that the EETS was an independent protective factor for posterior pituitary dysfunction (adjusted OR = 0.386, *p* = 0.04) and hypopituitarism (adjusted OR = 0.384, *p* = 0.031). The multivariate logistic regression analysis adjusted age, tumor size, sex, histopathology subtype, calcification, preoperative hypothalamic involvement, follow-up, and preoperative body mass index.

As shown in Table 3, gonadotropin deficiency was the most prevalent (24.8%) pre-existing hormone deficiency, followed by thyrotropin deficiency (21.4%), vasopressin deficiency (19.7%), growth hormone deficiency (12.0%), and adrenocorticotropic hormone deficiency (8.5%). Moreover, there were no significant differences between the EETS and TC groups in terms of pre-existing hormone deficiency, including anterior pituitary dysfunction, posterior pituitary dysfunction, ≥3 hormone deficiency, and hypopituitarism. During the follow-up, 52 new cases of anterior pituitary function worsening, 43 new cases of posterior pituitary dysfunction, 47 new cases of ≥3 hormone deficiency, and 48 new cases of hypopituitarism were observed in the whole cohort. The preliminary Chi-square test (Table 3) and further multivariate logistical analysis (Figure 3) confirmed that EETS was accompanied by lower frequencies of new-onset posterior pituitary dysfunction (uOR = 0.382, *p* = 0.024; aOR = 0.386, *p* = 0.040) and hypopituitarism (uOR = 0.388, *p* = 0.019; aOR = 0.384, *p* = 0.031), but not lower incidences of anterior pituitary function worsening (aOR = 0.503, *p* = 0.089) and new-onset ≥ 3 hormone deficiency (aOR = 0.517, *p* = 0.132).

### 3.4. Postsurgical Weight Change and Obesity

**Figure 4:**
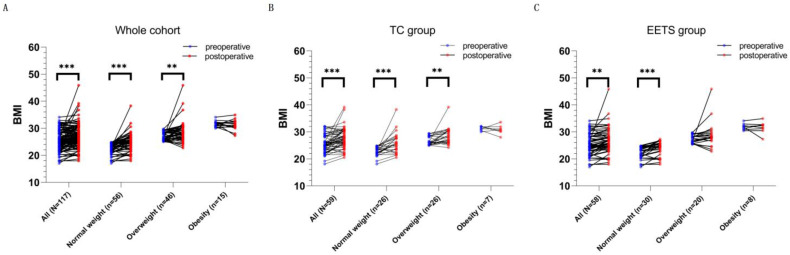
The preoperative and postoperative BMI based on the preoperative BMI category in the adult-onset craniopharyngioma cohort. (**A**) The difference between preoperative BMI and postoperative BMI in the whole cohort. (**B**) The difference between preoperative BMI and postoperative BMI in the TC subgroup. (**C**) The difference between preoperative BMI and postoperative BMI in the EETS subgroup. Compared to the TC group, the EETS group yielded similar impacts of the operation on weight change in patients with normal preoperative weight or preoperative obesity, except for patients with preoperative overweight. ** means *p* < 0.01, *** means *p* < 0.001.

The average BMI of the whole cohort increased from 25.50 kg/m^2^ preoperatively to 27.27 kg/m^2^ postoperatively (Table 4, Figure 4A, Appendix A Table A1, *p* < 0.001). After surgery, more than half of the patients experienced significant weight change (Table 4, considerable weight loss, *n* = 10, and considerable weight gain, *n* = 55). Patients who gained considerable weight outnumbered those who lost considerable weight, leading the number of patients with normal weight to decrease from 56 to 34 and the number of patients who were overweight or obese to increase from 61 preoperatively to 83 postoperatively. The increase in BMI of the whole cohort was mostly attributed to the increase in BMI of patients with normal weight (*p* < 0.001) or overweight (*p* < 0.001) since the BMI of patients with preoperative obesity did not increase significantly (Figure 4A, Appendix A Table A1). Stratified analyses were performed further to explore the impact of the surgeries on weight gain. EETS and TC showed a similar effect on patients with preoperative normal weight and preoperative obesity but a contrary effect on patients with preoperative overweight (Figure 4B,C, and Appendix A Table A1). Multivariate logistic regression analyses (Table 5) showed that the EETS was related to the lower rates for weight gain > 5% (aOR = 0.376, *p* = 0.034), significant weight change (aOR = 0.379, *p* = 0.022), and new-onset obesity (aOR = 0.259, *p* = 0.032). The present study found that age was associated with a higher likelihood of weight gain > 5% (aOR = 1.056, *p* = 0.007) and significant weight change (aOR = 1.055, *p* = 0.005). Furthermore, being preoperatively overweight was associated with a higher likelihood of postoperative obesity (aOR = 10.323, *p* < 0.001). Patients with preoperative obesity showed resistance to further weight gain (aOR = 0.029, *p* = 0.003) and significant weight change (aOR = 0.131, *p* = 0.006). Unfortunately, we failed to identify independent factors for weight loss > 5%.

## 4. Discussion

CP is a histologically benign tumor located in the intrasellar or suprasellar regions, and benign histology permits GTR to cure CP [21]. Hypothalamic damage caused by radical surgery may lead to severe complications that adversely affect survivors’ quality of life [22,23,24]. The relationship between surgery and clinical outcomes has been widely evaluated in cohorts of child-onset CP or mixed cohorts [11,12,13,14,25]. However, it still lacks consensus on the superiority of EETS to TC in the postoperative weight of CP, especially in the pure AOCP cohort. Therefore, we compared surgical, endocrinological, and weight-related outcomes among patients with AOCP who underwent EETS or TC to explore the superiority.

### 4.1. Surgical Outcomes

In EETS, the tumor is approached from below, potentially allowing better visualization of the tumor interface with the hypothalamus and undersurface of the optic chiasm and reducing retractions [26]. Moreover, the use of endoscopic techniques provides a larger field of view with excellent illumination, allowing for safe resection [26], which contributes to improved visual outcomes [11,12,14,25], lower rate of recurrence [11,12], less cranial nerve injury [13], and fewer postoperative seizures [11]. In addition to the surgical approach, preoperative obesity was also associated with GTR (Table 2). A higher HI level in obese patients before surgery makes achieving GTR difficult, which may account for the decreased rate of GTR in patients with preoperative obesity. Furthermore, Elliott et al. [5] reported that subtype (primary/recurrent), calcification, and tumor size were regarded as capable of affecting the extent of resection. In the current AOCP cohort, calcification and initial tumor size were not factors affecting the rate of GTR (Table 2). The EETS provides direct visualization to eliminate the risk of hypothalamic disruption and optic nerve injury from blind dissection, improves the ability to perform sharp extracapsular dissection of the tumor away from the visual apparatus and hypothalamus, and reduces damage to the hypothalamus and chiasm caused by tumor traction, which may theoretically contribute to the excellent ability of the EETS in safely resecting AOCP with giant size (Figure 2, case 2) and calcification (Figure 2, case 3) and overcoming the negative impacts of calcification and tumor size on the extent of resection.

The hypothalamic dysfunction caused by treatment or the tumor itself has been widely accepted as a cause of obesity and hyperphagia in CP [27,28]. Coincidentally, the EETS group had a higher rate of improved HI (Table 2) and a lower rate of new-onset obesity (Table 5) than the TC group. Furthermore, five patients developed worse HI only in the TC group (Table 1). To some extent, evidence from these two aspects supported the roles of EETS in hypothalamus sparing and eliminating further hypothalamic damage.

### 4.2. Endocrinological Outcomes

In the current study, we observed that pre-existing endocrine dysfunction appears to be permanent, and in fewer than 5% of patients with pre-existing endocrine dysfunction, endocrine function improved after surgery (data not shown). These findings are consistent with those reported by Patel et al. [29]. Additionally, we found that EETS can reduce the incidence of new hormonal deficits following surgery compared to TC, and the protective effects of EETS seem not to act on anterior pituitary function but only on posterior pituitary function and hypopituitarism (Figure 3).

Posterior pituitary function protection and DI management are necessary to prevent life-threatening electrolyte imbalances. Moreover, permanent DI was found at a higher rate in patients with aggressive surgery than in patients with less aggressive surgery combined with radiotherapy [30]. However, our team also favors the same view as that of Patel [29] and Yamada [31]: GTR but not STR should be the surgical goal for the first surgical attempt because it can prolong the time to tumor recurrence and reduce the need for postoperative adjuvant therapy. Moreover, under the premise of the EETS method, GTR was accomplished with a high rate of improved HI, less new-onset posterior pituitary dysfunction (Figure 3), and less new-onset hypopituitarism (Figure 3).

### 4.3. Weight Control and Obesity

Energy intake and expenditure are regulated by the ventromedial hypothalamus (VMH) and arcuate nucleus, which respond to peripheral satiety and hunger hormones, such as insulin, ghrelin, and leptin [32]. The damage to the VMH or arcuate nucleus predominantly contributes to extreme energy imbalance and progressive weight gain [33]. In addition to physical impairment, a study also found that adamantinomatous craniopharyngioma oily fluid may cause obesity by triggering inflammatory activation of microglia and damage to hypothalamus neurons [34]. Therefore, minimizing the damage to the hypothalamus caused by surgery is crucial for stable postoperative weight.

In the present cohort, the results confirmed that age contributes to weight gain, and EETS was related to a lower rate of weight gain > 5% following surgery (Table 5). Alternatively, patients with preoperative overweight in the TC group had an increased BMI, whereas the BMIs of patients with preoperative overweight were not increased in the EETS group, which indicates the more effective role of the EETS in postoperative weight control (Figure 4B,C, and Appendix A Table A1). Factors such as visual protection, surgical complications, length of stay, and tumor characteristics may influence the selection of surgical approaches, while our results provide some decision-making aid for the selection of surgical approaches in specific populations in terms of weight control. One population is patients who have serious concerns about weight gain and want to keep their body shape, especially models and women. Another population is patients with pre-existing obesity-related diseases, such as chronic obstructive pulmonary disease, arthritis, cardiovascular disease, and metabolism disease; the BMI increase could result in severe functional disability in these patients with pre-existing diseases [35,36]. Given the anxiety and deterioration of pre-existing diseases caused by weight gain, specific patients would prefer EETS for its advantage in weight control.

Regarding the relationship between postoperative weight gain and HI, a correlation between presurgical HI and postsurgical weight gain was observed in one study [37], and two other studies concluded that post-treatment weight gain is not associated with the pre- or postoperative hypothalamic assessment based on MRI [38,39]. Our conclusion is consistent with the latter: preoperative HI cannot predict weight gain (Table 5) or significant weight change (Table 5). Additionally, a previous pediatric study by Park et al. [40] demonstrated a correlation between the grade of preoperative HI and the prevalence of morbid obesity. Our study found that preoperative HI was associated with obesity at the last follow-up but not new-onset obesity (Table 5). Since 14 of 15 patients with pre-existing obesity excluded from multivariate analysis were of highest HI (grade 2), the rate of obesity of patients with grade 2 preoperative HI was significantly higher than that of patients with lower grade preoperative HI (29.3% for G2, 9% for G0 and G1, *p* = 0.011, data not shown). Though the length of follow-up also does not associate with weight gain or weight change (Table 5), one previous study found a significant increase in weight during the first three years after diagnosis, indicating that early therapeutic efforts on weight control should be considered for patients at risk for severe obesity [41].

Many previous studies have identified risk factors for obesity at the last follow-up in CP, including BMI at diagnosis [27,41], preoperative HI [27,41], maternal BMI [41], tumor size [42], and postoperative DI [43]. It is difficult to convert obese patients with CP into normal-weight individuals through current treatments; therefore, identifying the risk factors for new-onset obesity is of great clinical value in preventing lean patients from becoming obese by taking comprehensive and early measures. In the present study, TC and preoperative overweight were identified as independent factors for new-onset obesity following surgery in AOCP (Table 5), and older age was confirmed to be associated with weight gain. Therefore, these three factors help identify patients at risk for new-onset obesity or significant weight gain. Improving obesity awareness [44] and initiating early therapeutic efforts on weight control, including lifestyle interventions, pharmacotherapy, and bariatric surgery [1], are meaningful in preventing morbid obesity for AOCP patients at risk for new-onset obesity or weight gain.

Due to GTR and STR combined with radiotherapy having similar effects on the 5- and 10-year PFS or overall survival of CP [6], some surgeons advocate that limited surgical interventions combined with radiotherapy techniques seem to contribute to minimizing hypothalamic damage, especially in patients with CP adherent to the basal ganglia or the inferior surfaces of the hypothalamus. However, our experience suggests GTR as the goal of resection via EETS for several reasons, including but not limited to: (I) EETS improves the ability to perform more sharp extracapsular dissection of the tumor away from the hypothalamus with direct visualization of the surgical planes and increased illumination compared to the TC approaches, which contributes to an improved postoperative HI combined with a high rate of GTR (more than 90%, Table 2) and the lower occurrence of new-onset obesity (Table 5); (II) GTR can prolong the PFS; even the benign histology of CP permits cure by GTR [21]; (III) radiation significantly affects the intellectual level of patients, and hypothalamic radiation exposure is also a reported risk factor for hypothalamic obesity [45]. Therefore, we advocate GTR as the surgical goal, using EETS to reduce the need for postoperative adjuvant therapy and the occurrence of hypothalamic obesity.

### 4.4. Limitations

Our study has two main limitations. First, this study was retrospective, and the choice of surgical approach was still inevitably affected by many factors, such as individual surgeon preference, skill, experience, and tumor characteristics. Second, because the major increase in BMI occurred in the first three years after diagnosis, the follow-up period in our study was not long enough to assess and document a relatively static BMI at the end of the weight-change process and to observe the vast majority of positive outcomes in patients at risk for obesity. Therefore, randomized controlled trials with longer follow-up are necessary to address these limitations.

## 5. Conclusions

The EETS can result in higher rates of GTR and hypothalamus sparing in AOCP, as well as show advantages in protecting pituitary endocrine function and bodyweight control.

## Figures and Tables

**Table 1:** Preoperative characteristics and surgical outcomes of 117 patients with adult-onset craniopharyngioma. SD, standard deviation; IQR, interquartile range; ACP, adamantinomatous craniopharyngioma; BMI, body mass index; HI, hypothalamic involvement; TC, traditional craniotomy; EETS, endoscopic endonasal transsphenoidal surgery; GTR, gross total resection; STR, subgross total resection.

Preoperative Characterstics	Total Group (*n* = 117)	TC Group (*n* = 59)	EETS Group (*n* = 58)	*p*
Age at diagnosis (mean ± SD, year)	41.92 ± 15.51	41.02 ± 10.20	42.84 ± 12.74	0.393
Tumor size (mean ± SD, cm)	2.99 ± 0.95	2.89 ± 0.87	3.10 ± 1.03	0.245
Follow–up time (median, IQR, month)	26 (22–30)	26 (20–32)	25.50 (23–29)	0.724
Male sex	43 (36.7%)	22 (37.3%)	21 (36.2%)	0.904
Histopathology subtype (ACP)	72 (61.5%)	33 (55.9%)	39 (67.2%)	0.209
Calcification	77 (65.8%)	39 (66.1%)	38 (65.5%)	0.947
Preoperative BMI (mean ± SD, kg/m^2^)	25.50 ± 3.59	25.46 ± 3.96	25.54 ± 3.21	0.111
Preoperative BMI (category)				0.569
Normal weight	56 (47.9%)	26 (44.1%)	30 (51.7%)	
Overweight	46 (39.3%)	26 (44.1)	20 (34.5%)	
Obesity	15 (12.8%)	7 (11.8)	8 (11.8%)	
Preoperative HI				0.629
Grade 0	3 (2.6%)	2 (3.4%)	1 (1.7%)	
Grade 1	42 (35.9%)	19 (32.2%)	23 (39.7%)	
Grade 2	72 (61.5%)	38 (64.4%)	34 (58.6%)	
Surgical outcomes				
Extent of surgical resection				0.027
GTR	99 (84.6%)	46 (78.0%)	53 (91.4%)	
STR	18 (15.4%)	13 (22.0%)	5 (8.6%)	
Postoperative HI				<0.001
Grade 0	38 (32.5%)	18 (13.6%)	20 (34.5%)	
Grade 1	50 (42.7%)	17 (28.8%)	33 (56.9%)	
Grade 2	29 (24.8%)	24 (40.7%)	5 (8.6%)	
HI change				0.053
improved	77 (65.8%)	35 (59.3%)	42 (72.4%)	
no change	35 (29.9%)	19 (32.2%)	16 (27.6%)	
worse	5 (4.3%)	5 (8.5%)	0 (0%)	

**Table 2:** Independent factors for the extent of surgical resection and improved hypothalamic involvement. After adjusting for age, tumor size, sex, histopathology subtype, calcification, preoperative hypothalamic involvement, and preoperative body mass index, the EETS was independently associated with a higher rate of gross total resection (aOR = 4.08, *p* = 0.029) and improved hypothalamic involvement (aOR = 2.58, *p* = 0.041). uOR, unadjusted odd ratio; aOR, adjusted odd ratio; —, not report; * means *p* < 0.05.

Characteristics	GTR				HI Improvement			
	Unadjusted OR (95%CI)	*p*	Adjusted OR (95%CI)	*p*	Unadjusted OR (95%CI)	*p*	Adjusted OR (95%CI)	*p*
Age	1.01 (0.97–1.06)	0.686	1.03 (0.97–1.09)	0.403	0.99 (0.96–1.02)	0.59	0.99 (0.951–1.03)	0.584
Tumor size	1.03 (0.60–1.77)	0.928	1.02 (0.53–1.97)	0.946	1.45 (0.95–2.24)	0.09	1.12 (0.66–1.91)	0.672
Sex (female vs. male)	0.48 (0.15–1.58)	0.229	0.43 (0.11–1.62)	0.209	0.84 (0.38–1.86)	0.67	1.08 (0.42–2.73)	0.878
Histopathology subtype (ACP vs. PCP)	2.65 (0.93–7.58)	0.068	2.93 (0.85–10.07)	0.088	1.66 (0.77–3.61)	0.2	1.39 (0.55–3.52)	0.488
Calcification (yes vs. no)	1.06 (0.36–3.11)	0.917	1.05 (0.29–3.77)	0.947	1.00 (0.45–2.22)	0.99	0.78 (0.28–2.17)	0.64
Operation (EEA vs. TC)	3.82 (1.16–12.51)	0.027 *	4.08 (1.15–14.43)	0.029 *	1.93 (0.89–4.18)	0.1	2.58 (1.04–6.42)	0.041 *
Preoperative HI (G2 vs. G1&G0)	2.00 (0.71–5.64)	0.19	3.00 (0.82–11.05)	0.098	6.21 (2.70–14.32)	<0.001 *	8.86 (3.14–25.01)	<0.001 *
Preoperative BMI (normal weight, reference)	—	—	—	—	—	—	—	—
Overweight	1.37 (0.415–4.50)	0.608	1.18 (0.30–4.65)	0.814	1.24 (0.55–2.82)	0.61	0.74 (0.28–1.02)	0.561
Obesity	0.458 (0.12–1.80)	0.263	0.12 (0.02–0.81)	0.03 *	1.20 (0.36–3.99)	0.77	0.34 (0.07–1.60)	0.175

**Table 3:** Endocrine functions of the whole cohort. Pre-existing dysfunction is defined as dysfunction that occurred before surgery resection, and new-onset dysfunction is defined as dysfunction that occurred in patients without the same pre-existing dysfunction during follow-up. #, 23 patients with pre-existing posterior pituitary dysfunction were excluded from the analysis; $, 13 patients with pre-existing ≥ 3 hormone deficiency were excluded; &, 13 patients with pre-existing hypopituitarism were excluded. * means *p* < 0.05.

Pre-Existing Hormonal Outcomes	Total (*n* = 117)	TC Group (*n* = 59)	EETS Group (*n* = 58)	*p*
Anterior pituitary dysfunction				
Adrenocorticotropic hormone deficiency	10 (8.5%)	7 (11.9%)	3 (5.2%)	0.322
Thyroid-stimulating hormone deficiency	25 (21.4%)	12 (20.3%)	13 (22.4%)	0.827
Growth hormone deficiency	14 (12.0%)	7 (11.9%)	7 (12.1%)	1
Gonadotropic hormone deficiency	29 (24.8%)	11 (18.6%)	18 (31.0%)	0.121
Posterior pituitary dysfunction				
Antidiuretic hormone deficiency	23 (19.7%)	12 (20.3%)	11 (19.0%)	0.8517
≥3 hormone deficiency	13 (11.1%)	6 (10.2%)	7 (12.1%)	0.744
Hypopituitarism	13 (11.1%)	6 (10.2%)	7 (12.1%)	0.744
New-onset hormonal outcomes	Total	TC group	EETS group	*p*
Anterior pituitary function worsening	52 (44.4%)	30 (50.8%)	22 (37.9%)	0.161
Posterior pituitary dysfunction #	43 (45.7%)	27 (57.4%)	16 (34.0%)	0.024 *
≥3 hormone deficiency $	47 (45.2%)	27 (50.9%)	20 (39.2%)	0.231
Hypopituitarism &	48 (46.2%)	31 (58.5%)	18 (45.0%)	0.019 *

**Table 4:** The whole cohort’s body mass index and weight change. — means not reported.

Characteristics	Preoperative Record (Diagnosis)	Postoperative Record (Final Follow-Up)	*p*-Value
BMI	25.50 ± 3.59	27.27 ± 4.24	<0.001
BMI category			0.003
normal weight	56	34	
overweight	46	51	
obesity	15	32	
Weight change			—
weight loss > 5%	—	10	
weight stable	—	52	
weight gain > 5%	—	55	

**Table 5:** Independent factors for weight loss > 5%, weight gain > 5%, significant weight change, and postoperative obesity. The multivariate logistic regression analysis showed that EETS was an independent protective factor for weight gain > 5%, significant weight change, and new-onset obesity. #, the patients with stable weight were regarded as control events; 55 patients with weight gain > 5% were excluded. $, the patients with stable weight were regarded as control events, and 10 patients with weight loss > 5% were excluded. &, new-onset obesity was defined as obesity that occurred in patients without preoperative obesity during the follow-up; 15 patients with preoperative obesity were excluded. —, not reported; *, *p* < 0.05; **, *p* < 0.01; ***, *p* < 0.001.

Characteristics	Weight Loss > 5% #		Weight Gain > 5% $		Significant Weight Change		New-Onset Obesity &	
	Adjusted OR (95%)	*p*	Adjusted OR (95%)	*p*	Adjusted OR (95%)	*p*	Adjusted OR (95%)	*p*
Age	1.034 (0.965–1.108)	0.34	1.059 (1.016–1.103)	0.007 **	1.055 (1.016–1.095)	0.005 **	1.040 (0.990–1.091)	0.117
Tumor size	1.085 (0.399–2.950)	0.87	1.072 (0.651–1.765)	0.785	1.069 (0.667–1.713)	0.782	1.486 (0.765–2.887)	0.242
Follow-up	0.930 (0.794–1.088)	0.37	1.017 (0.932–1.111)	0.7	1.008 (0.930–1.093)	0.84	1.028 (0.927–1.14)	0.598
Sex (female vs. male)	0.394 (0.079–1.971)	0.26	0.697 (0.271–1.795)	0.454	0.577 (0.241–1.380)	0.216	1.073 (0.334–3.443)	0.905
Histopathology subtype (ACP vs. PCP)	0.472 (0.067–3.324)	0.45	1.323 (0.506–3.455)	0.568	1.142 (0.462–2.824)	0.773	1.420 (0.390–5.177)	0.595
Calcification (yes vs. no)	1.453 (0.212–9.982)	0.7	0.892 (0.328–2.426)	0.823	1.032 (0.410–2.593)	0.947	0.408 (0.108–1.543)	0.186
Operation (EETS vs. TC)	0.379 (0.079–1.971)	0.25	0.376 (0.152–0.930)	0.034 *	0.379 (0.165–0.870)	0.022 *	0.259 (0.075–0.892)	0.032 *
Preoperative HI (G2 vs. G1&G0)	0.693 (0.094–5.094)	0.72	1.395 (0.531–3.666)	0.5	1.293 (0.515–3.248)	0.585	2.534 (0.629–10.207)	0.191
Preoperative BMI (normal weight, reference)	—	—	—	—	—	—	—	—
Overweight	1.684 (0.204–13.938)	0.63	0.510 (0.195–1.334)	0.17	0.559 (0.227–1.381)	0.208	10.323 (3.646–29.227)	<0.001 ***
Obesity	3.312 (0.260–42.278)	0.36	0.029 (0.003–0.289)	0.003 **	0.131 (0.031–0.554)	0.006 **	— &	— &

## Data Availability

The data used in the current study are available from Yanbin Li and Youchao Xiao for reasonable use.

## Appendix A

**Table A1:** The preoperative and postoperative BMI based on the preoperative BMI category in the adult-onset craniopharyngioma cohort. Compared with the TC group, patients with preoperative overweight yield a stable postoperative BMI in the EETS group (preoperative BMI, 27.42 kg/m^2^; postoperative BMI, 28.93 kg/m^2^; *p* = 0.174, paired *t*-test). BMI, body mass index; TC, traditional craniotomy; EETS, endoscopic endonasal transsphenoidal surgery.

Preoperative BMI Category	ALL		*p* (Preoperative vs. Postoperative)	Normal Weight		*p* (Preoperative vs. Postoperative)	Overweight		*p* (Preoperative vs. Postoperative)	Obesity		*p* (Preoperative vs. Postoperative)
	Preoperative	Postoperative		Preoperative	Postoperative		Preoperative	Postoperative		Preoperative	Postoperative	
Whole cohort	25.50 ± 3.59	27.27 ± 4.24	<0.001	22.60 ± 2.06	24.83 ± 3.38	<0.001	27.03 ± 1.56	28.83 ± 3.98	0.003	31.62 ± 1.06	31.29 ± 1.96	0.465
TC group	25.46 ± 3.96	27.75 ± 3.72	<0.001	22.82 ± 1.77	25.90 ± 3.83	<0.001	26.73 ± 1.55	28.75 ± 3.06	0.003	31.23 ± 0.79	30.86 ± 1.71	0.596
EETS group	25.54 ± 3.21	26.60 ± 4.68	0.004	22.42 ± 2.29	23.90 ± 2.65	<0.001	27.42 ± 1.52	28.93 ± 5.02	0.174	31.97 ± 1.19	31.66 ± 2.19	0.652
